# Dental Miscellanies

**Published:** 1873-08

**Authors:** H. Scott

**Affiliations:** Lancaster, O.


### DENTAL MISCELLANIES.

BY H. SCOTT.

I cannot but feel gratified that Dr. Driscoll has permitted me to remain in existence, until after the meeting of the Mississippi Valley Association, in March. I had felt it a duty to publish my experience in the use of the oxychloride of zinc in caping, and generally, and did in connection with them, express my views somewhat boldly. I asked ten years trial for it, but only one or two have elapsed before a culmination seems approaching. I suppose it is allowable for one to express his gratification at being sustained in his theories and experiences and so I can do no more than to refer my friend to the discussion on the 251 page of the REGISTER of June, for the opinions of leading men of our profession on the use of the os artificiel.

### BREAKING TEETH.

The breaking, or crushing off of a tooth in attempting to extract, and failing at the same time to remove the roots, is at all times a sore annoyance, but doubly so when the pulp is left protruding. I suppose that every operator knows that accidents of the kind are liable to happen, when all the care has been observed that is possible, with the use of the best adapted instruments, in the most careful and skilled hands. Such an accident is most sadly perplexing, because there is not one patient out of ten who either can or will excuse the Dentist, but on the contrary will secretly feel, and not infrequently publish, that Dr. C— or D— broke a tooth for them, and in such a way too as not to disguise the dissatisfaction which, to others, conveys the impression that C— or D— is at least not skilled. I have seen many exceedingly perplexing cases of the kind, where I was compelled to split the roots and "dig" them out as well as I could, afterwards, and have known my patient on subsequent cases if tooth-ache occurred, to go to other offices. They are not to be blamed, but nevertheless it is humiliating. A molar not long since came half way out from its socket and then hung, while I tortured and racked it in all directions for several seconds, when it parted just above the bifurcation, and left both roots so firmly held in the bony arch, that for my life I could not get them away without separating, and then one of them fell back in the socket, and annoyed me ten minutes longer. A month afterwards the lady went to my neighbor and had a lateral incisor removed with great care, when she awarded him, the eclat of a fine tooth puller.

### THIRD FORMATION.

There is a lady residing in this community, aged a little over seventy years, who is wearing a full upper set on rubber. I believe it is about five years since she had her artificial denture. The case belonged to my partner, but I have known the lady intimately for many years. At the time the case was made, she had a fine upper mouth, with alveolar ridge very uniform. Within less than a year after the plate was set, she came back to say that a root had been left in her mouth, examination revealed a white glassy point protruding, at the place of the right cuspid. It was exceedingly firm and could not be shaken. It is there yet. It is a well formed cuspid, of about twice the size of the natural tooth, and much whiter than the original set, it has been repressed by the wearing of the rubber plate, and has not been projected far, the gum has wasted around it, and it is somewhat bare, but such are the fears of the old lady that she cannot be induced to have it taken away, though it interferes materially with the wearing of her plate.

### MANIA.

Can anything be done to counteract the growing mania everywhere found, of ladies, both single and married, insanely sacrificing pretty good natural teeth for the prettier ones the dentist can furnish? Who are to blame? I have met several young ladies recently who wanted their back teeth filled, but the front ones, though very little, decayed, in some instances. They said they were going to have them pulled out because they were "crooked and did not look well," and have a new set. So fixed were they in their minds on committing the sacrifice, that all my philosophy was wasted in trying to dissuade them. Such devotion to fashion is pitiful, and lies at the door of either the dental profession, or the leaders of fashion, or both. Such wicked and shameful pride leads to, and probably justifies the belief, that, should the project once be started, people will be found cutting off their pug and prominent noses in order to substitute better appearing ones.

### INTERESTING CASE.

A lady of about fifty years of age called at our office for advice some three weeks ago. The left side of her face was very much enlarged from the sub-orbit down to the neck, and was very firm and painful. Upon looking into the mouth it was found that the gums along the left lower side were very much enlarged, and discharging grumous fœted, saneous fluid, while two or three hard, jagged, and discolored points, were standing out above the surface. The probe discovered a large piece of exfoliated alveolar bone, which seemed to be held loosely by the super incumbent mass of diseased soft parts. Dr. Crider divided the gum, and with a stump forceps easily removed the exfoliated bone, which was honey combed, and exceedingly repulsive in every way. It proved to be the alveoli of the second bicuspid, and of the first and second molars. She had her teeth extracted on that side several years before, and had suffered greatly ever since ; and had been undergoing treatment at various times, and by various treatment, for the reduction of the swelling. Two or three subsequent incisions, with carbolic acid and other appropriate injections, by my son, J. C. Scott, were effectual. The lady is now well

Lancaster, O.

